# Feeding Management Strategies to Mitigate Methane and Improve Production Efficiency in Feedlot Cattle

**DOI:** 10.3390/ani13040758

**Published:** 2023-02-20

**Authors:** Michael L. Galyean, Kristin E. Hales

**Affiliations:** 1 Department of Veterinary Sciences, Texas Tech University, Lubbock, TX 79409, USA; 2 Department of Animal and Food Sciences, Texas Tech University, Lubbock, TX 79409, USA

**Keywords:** beef cattle, methane prediction, feeding management systems

## Abstract

**Simple Summary:**

Decreasing enteric CH_4_ emissions and nutrient excretion in feces and urine by growing and finishing beef cattle is an important climate-related goal for the beef industry. Feeding management systems such as programming cattle to achieve a specific rate of gain or restricting feed intake relative to predicted or observed ad libitum intake could be important tools to decrease overall feed intake and thereby decrease CH_4_ emissions and nutrient excretion. These management systems can increase the efficiency of gain relative to ad libitum feeding, but they can also increase time on feed, which offsets the effects of decreased feed intake. Using programmed feeding instead of traditional growing programs based on high-forage diets should decrease enteric CH_4_ emissions and nutrient excretion. For feedlot finishing, incorporating programmed or restricted feeding for a portion of the finishing period will have limited effects on CH_4_ emissions unless gain efficiency is increased during programming or in a subsequent ad libitum feeding period. Defining optimal feeding management systems that will reproducibly decrease enteric CH_4_ emissions and nutrient excretion should be the focus of future research.

**Abstract:**

Mitigation of greenhouse gases and decreasing nutrient excretion have become increasingly important goals for the beef cattle industry. Because feed intake is a major driver of enteric CH_4_ production and nutrient excretion, feeding management systems could be important mitigation tools. Programmed feeding uses net energy equations to determine the feed required to yield a specific rate of gain, whereas restricted feeding typically involves decreasing intake relative to the expected or observed ad libitum intake. In the context of growing/finishing systems typical of those in the United States and Western Canada, experimental results with programmed and restricted feeding have often shown decreased overall feed intake and increased gain efficiency relative to ad libitum feeding, but too much restriction can negatively affect harvest weight and associated carcass quality. Slick feed bunk management is a time-based restriction that limits day-to-day variation in feed deliveries, but the effects on intake and performance are not well defined. Simulations to estimate enteric CH_4_ emission and nitrogen excretion indicated that programmed feeding of a high-grain diet could appreciably decrease CH_4_ emissions and nitrogen excretion compared with traditional growing programs based on high-forage diets. For feedlot finishing, programming gain for a portion of the feeding period will decrease CH_4_ emission and N excretion only if cattle perform better than expected during the programmed phase or if compensatory growth occurs when cattle are transitioned to ad libitum feeding. Optimal approaches to implement programmed or restricted feeding that will yield increased efficiency should be the subject of future research in this area.

## 1. Introduction

Cattle are an important source of greenhouse gases (GHG) because of their ruminant digestive system. As a result, the potential adverse effects of beef cattle operations on the environment and climate change are a concern, and ways to mitigate CH_4_ emissions from cattle feeding operations are an important practical research issue for the industry. As a component of mitigation strategies, common cattle feeding practices need to be evaluated and balanced for production efficiency and environmental concerns. The primary GHG associated with animal feeding are CH_4_ and N_2_O, which are produced both directly from cattle (i.e., enteric emission of CH_4_) and indirectly from nutrients excreted in the urine and feces for N_2_O. Methane has 21 to 26 times the global warming potential (GWP) of carbon dioxide, and nitrous oxide has 296 to 310 times the GWP of carbon dioxide [1]. Methane emissions from feedlot cattle arise primarily from ruminal fermentation [2], whereas N_2_O is formed during nitrification and denitrification from nitrogenous compounds present in the feces and urine of cattle [3]. Thus, excreted nutrients in urine and feces have an indirect effect on GHG emissions from the feedlot pen surface [4,5,6]. Urinary N excretion also influences ammonia emissions from the feedlot surface [7], which can have negative environmental consequences. Because feed intake drives both enteric emissions and nutrient excretion, precision management of feed intake could have important effects on subsequent indirect emissions of GHG and ammonia.

In the present review, we will define various feeding management strategies and describe their effects on production efficiency in feedlot cattle. Our focus will be on reviewing studies that have evaluated the effects of feeding management strategies on production in growing/finishing systems for feedlot beef cattle typical of those in the United States and Western Canada. Based on the expected changes in feed intake, nutrient excretion, and animal performance, we will also project the potential effects of these feeding systems on enteric CH_4_ emissions and N excretion.

## 2. Defining Feeding Management Strategies

Based on a previous review [8], definitions were developed for two primary feeding management strategies—programmed feeding and restricted feeding. Programmed feeding was described as a method that uses net energy equations [9] to determine the quantity of feed required for a specific average daily gain (ADG; [8]). Multiple options within restricted feeding were described, which included any method of managing feed intake that results in restriction relative to actual or expected feed intake. Restriction could be in the form of altering the quantity of feed provided or the time of access to feed. Intensive bunk-reading and feed-delivery systems (clean or slick bunk management) are typically based on time restriction [8], with the potential to affect feed intake relative to ad libitum access depending on how these systems are applied in practice.

Why would producers of growing–finishing beef cattle apply these feeding management systems? Providing an alternative to pasture-based systems for growing cattle, improved feed efficiency, and decreased production of manure are key elements related to the decision to use these systems. Other aspects include avoiding the over-consumption of feed, decreasing the variation in feed intake, simplified feed bunk management and feed milling and delivery logistics, identification of sick cattle (primarily with programming at a lower ADG), and providing a system from transitioning cattle to ad libitum consumption of high-concentrate finishing diets [8]. In the following sections, we will focus on the effects of precision feeding systems on changes in feed intake and the efficiency of production.

## 3. Effects of Feeding Management on Animal Performance and Production Efficiency

Feed represents 65 to 75% of the cost in beef production [10]. In addition, feed is used with a relatively low efficiency [11], with beef cattle recovering less than 20% of gross energy consumed across most diets [12,13,14,15,16]. Optimized precision animal management is achieved by providing the nutrients an animal needs without over-feeding nutrients. Both restricted and programmed feeding can improve feed efficiency [8], which presumably relates to the observation that ADG increases at a decreasing rate as feed intake increases [11], but the magnitude of the effects of these feeding management strategies on efficiency has been variable in the literature.

### 3.1. Production Responses with Programmed Feeding

Programmed feeding, particularly with relatively high-concentrate diets, has been most frequently evaluated as an alternative to traditional growing programs based on higher-roughage diets of low-to-moderate energy concentration. The impetus for research in this area is often driven by the high cost or low availability of traditional roughage sources, but the ancillary benefits of programming gain with high-concentrate diets such as consistency in feed delivery requirements and associated logistics, as well as the potential for compensatory gain when intake is increased to ad libitum, can be important considerations that drive field application. Several experiments on programmed feeding have been conducted by researchers at the Ohio State University. In the first of two experiments [17], ad libitum feeding was compared with various programmed-gain strategies (different rates and patterns of gain for various periods of time), followed by ad libitum access to feed until harvest. Overall, observed programmed ADG was greater than predicted from net energy equations, and systems that decreased ADG for longer periods of time resulted in less total feed consumption to reach equivalent harvest endpoints. Similarly, Loerch and Fluharty [18] compared ad libitum access to feed with different programmed rates and patterns of gain followed by ad libitum access. As with the previous work [17], observed ADG exceeded predicted ADG, and programming for stepwise increases in ADG followed by approximately 100 d of ad libitum feeding improved feed efficiency and decreased total feed consumption by more than 100 kg/steer, with no effects on carcass weight and quality. In a third report from Ohio researchers [19], continuous ad libitum feeding was compared with programmed gain for the first 63 to 66 d on feed at approximately 68 to 78% of ADG achieved by ad libitum-fed cattle, followed by programming at an increased ADG for the next 60 to 70 d, with a final period of ad libitum feeding. Programmed feeding improved gain efficiency and decreased total feed consumed compared with ad libitum-fed cattle by approximately 5 to 9%, without any major effects on carcass characteristics.

Steers programmed to gain 1.4 kg/d for the first 62 d of a finishing period followed by ad libitum feeding did not perform better than expected from net energy calculations [20], but they tended to have greater gain efficiency than steers given ad libitum access to feed for the entire period. Across implanted vs. non-implanted treatments, programming gain decreased total feed intake during the feeding period by an average of 124.6 kg. Felix et al. [21] programmed gain at 0.9 or 1.4 kg/d for a 98-d growing period, with diets based on corn or dried distillers grains plus solubles (DDGS), followed by an ad libitum finishing period. Programmed gain was greater than expected with corn-based diets but not with DDGS diets. Gain efficiency for the overall feeding period did not differ because of programmed ADG, nor did total feed intake, and programmed ADG did not affect digestibility of dry matter, neutral detergent fiber, and ether extract.

In two experiments [22], steers were fed different intakes of diets that varied in energy concentration to achieve an ADG of 1.6 kg/d. As expected, the diet with the greatest energy concentration resulted in the greatest gain efficiency, but it also resulted in greater ADG, despite similar formulated intakes of net energy. In a companion study [23], the digestibility of the diets differing in energy concentration that were fed to yield an ADG of 1.6 kg/d was evaluated. The diet with the highest energy concentration had an increased digestibility of DM, and it increased the ruminal proportion of propionate, reflecting the greater starch and lower fiber concentrations in the diet, resulting in a lower calculated loss of CH_4_ and a greater ME concentration with the higher-energy diet. Thus, diets with higher energy concentrations are likely optimal for programmed feeding, particularly if decreasing the environmental footprint of cattle feeding is a goal. As noted previously, higher-energy diets during programmed-gain periods also facilitate the transition to ad libitum feeding of a high-concentrate diet.

The physiological responses to programmed feeding have not been fully elucidated, but the effects on maintenance requirements of a relatively constant feed intake to yield a fixed ADG might explain improved feed efficiency with programmed feeding [24,25]. In addition, compensatory gain is often noted during ad libitum feeding of cattle previously programmed at low rates of gain, which presumably contributes to improved overall gain efficiency with this feeding management strategy.

### 3.2. Production Responses with Restricted Feeding

In research studies, restricted feeding is typically an approach in which feed intake is restricted relative to cattle with ad libitum access to feed. Obviously, intake by ad libitum-fed cattle varies from day to day and is affected by environmental and management factors, so comparisons are often made to average ad libitum intake for a previous period (e.g., the average of the previous week). Because the real-time application of restriction relative to ad libitum intake is challenging, there has been limited use of comparative restriction in practice. Thus, if implemented in feedlots, restriction is most likely applied relative to anticipated or predicted ad libitum intake. The challenge with the practical application of restricted feeding might have contributed to the development of time restriction approaches such as slick or clean bunk management, which are more easily applied in feedlots. Despite its practical challenges with application, however, comparative restricted feeding has been a useful research technique.

Hicks et al. [26] conducted some of the earlier research on restricted feeding. In two experiments, intake was restricted (85 or 89%) relative to ad libitum intake, whereas in a third experiment, intake was restricted to 80% of ad libitum for the first 56 d of a 138 d study followed by ad libitum access, or gain was programmed at 1.35 or 1.5 kg/d. Restriction improved feed efficiency relative to ad libitum feeding, but carcass quality grade was decreased by restricted feeding or programming gain. The digestibility of dry matter and starch was not affected by restriction or programming. The authors suggested that limiting day-to-day variation in feed intake could be a significant advantage of controlled feeding systems.

Restricted feeding of a high-grain diet at 80% of the intake achieved with ad libitum feeding of a traditional corn silage-based growing diet for 77 d, followed by ad libitum access to a high-concentrate finishing diet to harvest at 149 d, decreased intake for the overall feeding period and improved feed efficiency [27]. Restricting intake of an all-concentrate diet to 80 or 90% of ad libitum resulted in a linear improvement in feed efficiency but increased days on feed to achieve a similar final weight [28]. Moreover, carcass fat was decreased linearly with restriction vs. ad libitum feeding. In a second experiment, restricting intake of a silage-based growing diet to 80 or 90% of ad libitum for 84 d followed by ad libitum access until harvest resulted in a linear improvement in feed efficiency and a linear decrease in total feed intake for the overall feeding period (42 and 135 kg for the 90% and 80% restrictions, respectively; [28]). Feed restriction decreased carcass fatness and quality grade. Similarly, decreased fat cover in cattle fed barley- or corn-based diets restricted to 96% of ad libitum has been reported [29], but restriction did not improve feed efficiency relative to ad libitum feeding.

The degree of restriction could be important in terms of feed efficiency and carcass characteristics. Among groups restricted to 95%, 90%, and 85% relative to the dry matter intake (DMI) of ad libitum-fed cattle [30], optimal feed efficiency was observed for the 90% restriction (quadratic response). Fat thickness and marbling score were least for the 85% restriction, suggesting that carcass quality is likely to be affected negatively by more severe restrictions in feed intake.

Silva et al. [31] noted that the length of restriction is an important component of the potential environmental benefits of feed restriction. Restriction to 85% of ad libitum intake for 28, 42, or 84 d followed by ad libitum feeding to yield a total of 84 d on feed did not affect digestibility of dry matter, and fecal phosphorus excretion measured near the end of the feeding period was decreased only for the 84-d restriction. A 75% restriction of a grower diet based on alfalfa, sorghum silage, and modified distillers grains plus solubles decreased absolute CH_4_ production (g/d), but did not affect CH_4_ production per unit of DMI [32]. Although the environmental effects of restricted (and programmed) feeding systems can be estimated from feed intake data and assumptions about digestibility, more direct experimentation to measure the digestibility of nutrients and CH_4_ emissions would be beneficial.

### 3.3. Production Responses with Feed Bunk Management Systems

Commercial feedlots in the U.S. and Canada frequently use intensive feed bunk management approaches during the feedlot finishing period. These systems typically involve observations of feed bunks to determine when the bunks are “slick” or “clean” (hence the terminology slick or clean bunk management). Such systems commonly have a target for a time when the feed bunk is empty, which is monitored with a bunk-scoring system. The target can range from a full 24-h cycle to several hours less than a 24-h cycle and be modified by the bunk score (e.g., completely slick vs. some small amount of feed). To avoid restricting intake relative to ad libitum, these systems also normally include a challenge approach, such that when a pen of animals meets the target for a 3-d period, the total quantity of feed is increased by a small amount (e.g., 0.1 kg/animal).

A key objective of slick bunk management is to limit the day-to-day variation in feed consumed by the pen. This is clearly an achievable goal, but whether this approach decreases intake variation by individual animals within the pen, particularly if intake is not restricted, is open to question. Even if there are no performance or animal health advantages with slick bunk management, the system clearly offers advantages in feeding logistics and milling by bringing more consistency and predictability to daily feed deliveries, which might explain its relatively high level of adoption by the U.S. feedlot industry.

Few studies have evaluated the effects of controlled bunk management systems on performance and carcass characteristics of feedlot cattle, and results have been variable. This variability could reflect how slick bunk management is applied at a given location and particularly the degree of restriction that might be imposed. In their review of feed intake variation and bunk management, Pritchard and Bruns [33] reported the results of an experiment in which intake was managed to eliminate extreme swings in feed intake that are frequently noted with ad libitum feeding. The managed approach decreased overall feed intake by 12% compared with ad libitum feeding, while eliminating highs and lows in intake over time. The ADG was not changed but was more variable among ad libitum-fed cattle, and gain efficiency was significantly improved by managed feeding. Carcass weight and marbling score did not differ between ad libitum and managed cattle.

In steers fed twice daily (60% at 0700 h and 40% at 1130 h), with feed bunks managed to achieve targets of 0, 0.25, or 1 kg of feed in the bunk 30 min before feed delivery [34], DMI decreased from 11.2 to 10.4 and 9.7 kg for the bunk targets of 1, 0.25, and 0 kg, respectively. This study was conducted as a Latin square design with 10 d adaptation and 4 d data collection periods, so the extent to which these data are applicable to long-term use of slick bunk management is open to question. Nonetheless, the results suggest the potential for at least short-term restriction of feed intake with more aggressive slick bunk management.

In contrast to the findings of [34], Smock et al. [35] found no effect of slick bunk management on ADG, DMI, and carcass characteristics in cattle fed steam-flaked corn-based diets for an average of 177 d. The slick bunk management system in this study targeted from 0 to 2% of the previous day’s feed delivery remaining in the bunk, whereas the target for ad libitum-fed cattle was 5% remaining in the bunk. For slick bunk cattle, the target was achieved 55% of the time, with the target for the ad libitum cattle achieved 59% of the time. Arguably, feed deliveries for both treatments in this study were managed, but it seems clear that slightly more intensive management of feed deliveries did not affect feed intake.

Because of the limited research that has been conducted on intensive bunk management systems, it is difficult to draw definitive conclusions about how these strategies affect feed intake and performance. Part of the challenge with research in this area is that the application of slick bunk management is very dependent on the personnel who are applying the system. Thus, the extent to which research findings can be broadly applied to the feedlot industry is a concern.

## 4. Mitigation of Greenhouse Gases through Feeding Management

Research has provided numerous enteric CH_4_ mitigation strategies, such as alternative electron receptors, CH_4_ inhibitors, dietary lipids, and increased animal production efficiency [36]. Methane production in the rumen is an evolutionary adaptation that allows the rumen to dispose of hydrogen ions (H+), a fermentation byproduct and an important energy substrate for methanogenic bacteria. Without the ability to dispose of it, H+ would accumulate, decrease ruminal pH, and inhibit carbohydrate fermentation and fiber degradation [36]. Although several compounds are effective in decreasing enteric CH_4_ emissions, they can also decrease feed intake, fiber digestibility, and animal productivity, and ruminal microbes might adapt to these compounds in various ways [37]. Nonetheless, Beauchemin et al. [37] noted that many of the feed additive approaches to mitigate CH_4_ are compatible with other mitigation approaches such as dietary alterations or feeding and grazing management.

As feed intake by cattle increases, CH_4_ production also increases because more substrate is available for fermentation. Indeed, feed intake has the greatest effect on CH_4_ production of any measurable production characteristic [9]. Greater ADG with increased feed intake can result in decreased CH_4_ per unit of product, but absolute CH_4_ production would be expected to increase with increased feed intake. The goal of applying feeding management systems as a mitigation tool would be to decrease absolute CH_4_ emissions, while not changing or decreasing CH_4_ per unit of beef produced (e.g., CH_4_/kg of carcass weight). Using feeding management strategies to decrease intake overall or during a portion of the feeding period, while achieving an equivalent or superior feed efficiency at equal carcass weight and quality, could have an appreciable effect on absolute CH_4_ production and the carbon footprint of beef feedlots. Moreover, because N_2_O emissions are primarily a function of feces and urine excreted onto the feedlot surface, decreased feed intake associated with feeding management systems should decrease total nutrients excreted during the feeding period, thereby decreasing N_2_O emissions from the feedlot surface.

In the following sections, we will use simulated feeding scenarios to estimate the extent to which programmed-feeding approaches could affect enteric CH_4_ emissions and N excretion associated with feedlot cattle production. We will focus on programmed feeding because feedlots are unlikely to apply restricted feeding in practice, other than to restrict intake relative to predicted or historically observed estimates of ad libitum intake, which results in a practical application of the method being similar to programmed feeding. Two scenarios with programmed feeding will be considered: (1) use of programmed feeding as an alternative to traditional high-forage growing programs before a feedlot finishing period; (2) use of programmed feeding for a portion of the feedlot finishing period.

### 4.1. Potential to Mitigate Methane and Nitrogen Excretion with Programmed Feeding during a Growing Program before Feedlot Finishing

Projected responses to three different growing programs followed by a common feedlot finishing period are shown in Table 1. The use of a corn silage-based diet in a drylot and grazing annual winter wheat forage are commonly used programs in the U.S. to grow weaned calves to a heavier body weight (BW) before placement in a feedlot, particularly in the Midwest and Great Plains. We compared these two traditional practices to programmed feeding with a high-concentrate diet, with the ADG of programmed cattle set to equal the ADG expected with the corn silage-based program. Hence, the days on feed for the silage and programmed-gain systems were equal, whereas the grazing days for cattle on wheat pasture, which had a lower ADG than silage-fed cattle, were necessarily longer to achieve the same BW at the start of the feedlot finishing phase. Feed intake as a percentage of BW for the corn silage and wheat forage groups was predicted from the equations of [9], and diet composition data (starch, crude protein, ether extract, neutral detergent fiber, and ash) were calculated from tabular values [9]. Gain was projected or programmed using net energy equations [9], and enteric CH_4_ production was calculated using the starch:neutral-detergent-fiber ratio and ether extract equation of [38], as well as the IPCC Tier 2 [39] approach. Nitrogen excretion was estimated from N intake minus N retained, which was calculated using the net protein in the gain calculation of [9].

Relative to programmed gain (set at 100%), both a corn silage-based growing program (126%) and wheat pasture grazing (187%; based on the equation of [38]) followed by an ad libitum feedlot finishing period substantially increased enteric CH_4_ production. For the silage program, the increased CH_4_ emissions reflect greater DMI, as well as greater fiber and lower starch concentrations vs. the programmed-gain diet, whereas the increase with wheat pasture grazing reflects greater DMI, dietary effects, and a longer growing period. Nitrogen excretion increased slightly for the silage growing program (105%) vs. programmed gain, reflecting the greater DMI, with a substantial increase in N excretion vs. programmed gain for wheat pasture grazing (146%). The increase in N excretion with wheat pasture grazing was expected because of a longer growing period and a greater dietary N concentration than the other programs. Based on this simulation, the use of a high-concentrate diet in a programmed-feeding approach during a growing period would be expected to decrease enteric CH_4_ emissions, and potentially decrease N excretion, vs. traditional programs.

### 4.2. Potential to Mitigate Methane and Nitrogen Excretion with Programmed Feeding during a Portion of Feedlot Finishing

The same methods used for estimating enteric CH_4_ emissions and N excretion in the growing phase were applied to the comparison of three feedlot finishing scenarios—continuous ad libitum access to feed (the standard practice) vs. programmed feeding at two different rates of gain (80 or 90% of the ADG predicted for ad libitum-fed cattle) for a portion of the feeding period (Table 2). The portion chosen for programmed gain was from an initial BW of 360 kg to an intermediate BW of 500 kg, followed by allowing the cattle ad libitum access to feed from 500 kg to the final BW of 610 kg. The same diet was assumed to be fed to all groups.

Relative to continuous ad libitum feeding, both programmed-gain scenarios slightly increased enteric CH_4_ emissions over the entire feeding period (104 and 101% of ad libitum for the 80 and 90% of ad libitum ADG programmed-gain groups, respectively, using the equation of [38]). These increases in CH_4_ reflect the increased total days on feed for the programmed-gain groups to achieve a final BW equal to ad libitum-fed cattle (173 and 161 total days on feed for the 80 and 90% ADG programmed groups, respectively, vs. 155 days for the ad libitum group). Because this simulation did not assume any increases in efficiency of gain or compensatory gain responses in programmed-fed cattle, we considered the possibility of a compensatory response in feed intake (a 7.5% increase) when programmed-gain cattle were shifted to ad libitum feeding at a BW of 500 kg (Table 2). Allowing for this compensatory response, which increased gain efficiency by 3.4% during the final ad libitum feeding period for programmed-gain cattle, the relative changes in enteric CH_4_ emissions were decreased to 102 and 99% of the continuous ad libitum cattle for the 80 and 90% ADG groups, respectively. Results of previous experiments (see Section 3.1) suggest that increased gain efficiency of the magnitude we assumed or greater is a frequent observation when programmed cattle are switched to ad libitum feeding. Moreover, based on our review of data from programmed-feeding studies, ADG during the programmed-feeding period is often greater than expected.

## 5. Summary and Recommendations for Further Research

Our simulations suggest that the most likely option for using programmed or restricted feeding to decrease enteric CH_4_ emissions would be as a replacement for traditional growing programs such as silage-based grower diets or grazing of higher-quality forages. For cattle fed to a common final BW, decreasing enteric CH_4_ emissions by using programmed or restricted feeding during feedlot finishing requires increased gain efficiency with programmed feeding during the programmed-gain phase, the subsequent ad libitum feeding period, or both. It should also be noted that the extent to which the overall carbon footprint of various systems would differ requires a much more extensive simulation than we conducted, including production and sourcing of dietary ingredients, manure management, overall fossil fuel use, etc. Moreover, the economic aspects of these production systems should also be considered in defining their ultimate sustainability.

Based on our review of the literature and simulations, precision feeding management systems such as programmed and restricted feeding seem to have the potential to increase gain efficiency, and with that improved efficiency and associated lower feed intake, decrease enteric CH_4_ emissions. For replacement of traditional high-forage growing programs, much of the benefit of programmed feeding with a high-concentrate diet is associated with a lower DMI and increased starch concentration in the diet, both of which decrease enteric CH_4_ emissions. Confirmatory field-based estimates of CH_4_ emissions when programmed feeding is used in growing programs are needed, but this approach seems highly likely to be a useful tool in decreasing emissions in situations where it makes sense economically and logistically. For the feedlot finishing period, increased gain efficiency with programmed and restricted feeding should decrease CH_4_ emissions but, at present, we lack an understanding of the best approaches to apply such feeding systems in a manner that will result in no change in carcass quality and economics of production. When ad libitum feeding is used after a period of programmed or restricted feeding, defining the optimal length of the programmed- or restricted-feeding period relative to the entire feedlot finishing period is a necessary step, as is defining the optimal ADG during the programmed-feeding period. In addition, determining whether programming ADG at a high level (e.g., near projected or historically observed ADG for similar cattle) for the entire feedlot finishing period can be successfully used to finish cattle to an appropriate harvest BW and carcass quality is an important research question. Again, field-based estimates of CH_4_ emissions, as well as evaluation of nutrient excretion and manure production, should be a component of future research programs in this area.

Overall, feeding management strategies such as programmed- and restricted-feeding programs offer considerable practical benefits to beef producers, including logistical advantages in planning feed resource allocation and delivery, animal health management, and the potential for increased gain efficiency, leading to decreased feed intake and thereby decreased enteric CH_4_ emissions and nutrient loads in the environment. Refining these systems to decrease the environmental footprint of the cattle feeding industry should be an important research objective in the years ahead.

## Figures and Tables

**Table 1:** Effects of traditional high-roughage growing programs vs. a programmed-gain high-concentrate growing program followed by an ad libitum finishing period on estimated enteric emission of methane and excretion of nitrogen in feedlot beef cattle.

	Growing Program			
Item ^1^	Silage Grower Diet	Programmed-Gain High-Concentrate Diet	Wheat Forage Grazing	Ad Libitum Finishing Period
Initial BW, kg	250	250	250	360
Ending BW, kg	360	360	360	610
Days fed	84	84	136	155
DMI, kg/d	6.80	5.85	7.51	9.19
ADG, kg	1.30	1.30	0.81	1.62
Diet				
Starch, %	37.35	60.66	4.11	63.41
NDF, %	34.66	16.03	54.16	13.60
EE, %	3.34	3.65	3.00	5.44
CP, %	12.39	12.43	15.32	13.61
CP from NPN, %	1.41	1.43	1.21	2.25
Ash, %	5.26	3.63	8.91	4.20
GE, Mcal/kg ^2^	4.30	4.38	4.17	4.45
NEm, Mcal/kg	1.84	2.09	1.37	2.15
NEg, Mcal/kg	1.21	1.43	0.79	1.48
Total CH_4_, g/d ^3^	132.8	73.0	167.4	83.4
Total CH_4_, g/d ^4^	143.9	58.3	154.3	93.0
Total N excreted, g/d ^5^	100.2	81.8	161.9	168.9
Relative change in CH_4_, % ^6^	126	100	187	-
Relative change in N, %^6^	105	100	146	-

^1^ BW = shrunk body weight; DMI = dry matter intake; ADG = average daily gain; NDF = neutral detergent fiber; EE = ether extract; CP = crude protein; NPN = non-protein nitrogen; GE = gross energy; NEm = net energy for maintenance; NEg = net energy for gain. The DMI for all groups other than the programmed-gain high-concentrate diet was calculated using Equation (10-2) from [9]. ^2^ Calculated using Equation (2) from [40]. ^3^ Calculated using Equation (2) from [38]. ^4^ Calculated using IPCC Tier 2 [39] equations (coefficients of 0.065 and 0.030 for <90% and >90% concentrates, respectively). ^5^ Calculated as total N intake minus N retained determined from the net protein in gain equation (Equations (19)–(51)) of [9]. ^6^ Change in CH_4_ (from the equation of [38]) and total N excretion, based on the entire feeding period, relative to the programmed-gain high-concentrate diet.

**Table 2:** Effects of programmed feeding a high-concentrate diet at two different rates of gain followed by an ad libitum finishing period vs. continuous ad libitum feeding on estimated enteric emission of methane and excretion of nitrogen in feedlot beef cattle.

Item ^1^	Programmed at 80% of Ad Libitum ADG	Programmed at 90% of Ad Libitum ADG	Ad libitum Feeding Period after Programmed Gain with No Compensatory Response	Ad Libitum Feeding Period after Programmed Gain with a Compensatory Response	Continuous Ad Libitum Feeding
Initial BW, kg	360	360	500	500	360
Ending BW, kg	500	500	610	610	610
Days fed	108	96	65	58	155
DMI, kg/d	7.31	7.85	10.51	11.30	9.19
ADG, kg	1.29	1.45	1.70	1.89	1.62
Diet					
Starch, %	63.41	63.41	63.41	63.41	63.41
NDF, %	13.6	13.6	13.6	13.6	13.6
EE, %	5.44	5.44	5.44	5.44	5.44
CP, %	13.61	13.61	13.61	13.61	13.61
CP from NPN, %	2.25	2.25	2.25	2.25	2.25
Ash, %	4.20	4.20	4.20	4.20	4.20
GE, Mcal/kg ^2^	4.45	4.45	4.45	4.45	4.45
NEm, Mcal/kg	2.15	2.15	2.15	2.15	2.15
NEg, Mcal/kg	1.48	1.48	1.48	1.48	1.48
Total CH_4_, g/d ^3^	66.3	71.2	95.4	102.5	83.4
Total CH_4_, g/d ^4^	73.9	79.4	106.4	114.4	93.0
Total N excreted, g/d ^5^	131.0	139.6	200.7	215.2	168.9
Relative change in CH_4_, no compensation % ^6^	104	101	-	-	100
Relative change in CH_4_, with compensation, % ^6^	102	99	-	-	100
Relative change in N, no compensation, % ^6^	104	101	-	-	100
Relative change in N, with compensation, % ^6^	102	99	-	-	100

^1^ BW = shrunk body weight; DMI = dry matter intake; ADG = average daily gain; NDF = neutral detergent fiber; EE = ether extract; CP = crude protein; NPN = non-protein nitrogen; GE = gross energy; NEm = net energy for maintenance; NEg = net energy for gain. The DMI for all groups other than the programmed-gain high-concentrate diet was calculated using Equation (10-2) from [9]. ^2^ Calculated using Equation (2) from [40]. ^3^ Calculated using Equation (2) from [38]. ^4^ Calculated using IPCC Tier 2 [39] equations (coefficients of 0.065 and 0.030 for <90% and >90% concentrates, respectively). ^5^ Calculated as total N intake minus N retained determined from the net protein in gain equation (Equations (19)–(51)) of [9]. ^6^ Change in CH_4_ (from the equation of [38]) and total N excretion, based on the entire feeding period, relative to continuous ad libitum feeding with or without compensation after programmed feeding.

## Data Availability

Data sharing is not applicable to this article.
